# Paraoxonase/Arylesterase Activity of Serum Paraoxonase-1 and Schizophrenia: A Systematic Review and Meta-Analysis

**DOI:** 10.3390/antiox12081484

**Published:** 2023-07-25

**Authors:** Angelo Zinellu, Stefania Sedda, Arduino A. Mangoni

**Affiliations:** 1 Department of Biomedical Sciences, University of Sassari, 07100 Sassari, Italy; azinellu@uniss.it (A.Z.); s.sedda4@studenti.uniss.it (S.S.); 2 Discipline of Clinical Pharmacology, College of Medicine and Public Health, Flinders University, Bedford Park, SA 5042, Australia; 3 Department of Clinical Pharmacology, Flinders Medical Centre, Southern Adelaide Local Health Network, Bedford Park, SA 5042, Australia

**Keywords:** schizophrenia, paraoxonase-1, paraoxonase, arylesterase, oxidative stress, HDL cholesterol, cardiovascular disease, atherosclerosis

## Abstract

The presence of a pro-oxidant state in patients with schizophrenia may account for the increased risk of atherosclerosis and cardiovascular disease in this group and supports the potential utility of circulating biomarkers of oxidative stress for risk stratification and management. We investigated this issue by conducting a systematic review and meta-analysis of the association between the circulating concentrations of paraoxonase-1, an antioxidant calcium-dependent high-density lipoprotein (HDL)-associated esterase, with paraoxonase and arylesterase activity in schizophrenia. We searched electronic databases from inception to 31 May 2023 for studies investigating paraoxonase-1 in patients with schizophrenia and healthy controls and assessed the risk of bias and the certainty of evidence (PROSPERO registration number: CRD42023435442). Thirteen studies were identified for analysis. There were no significant between-group differences in paraoxonase (standard mean difference, SMD = 0.12, 95% CI −0.23 to 0.48, *p* = 0.50; extremely low certainty of evidence) or arylesterase activity (SMD = −0.08, 95% CI −0.39 to 0.23, *p* = 0.61; very low certainty of evidence). However, in meta-regression and subgroup analysis we observed significant associations between the SMD of paraoxonase and age (*p* = 0.003), HDL–cholesterol (*p* = 0.029), and study country (*p* = 0.04), and the SMD of arylesterase and age (*p* = 0.007), body mass index (*p* = 0.012), HDL–cholesterol (*p* = 0.002), and pharmacological treatment for schizophrenia (*p* < 0.001). In the absence of overall between-group differences, our systematic review and meta-analysis suggests that alterations in paraoxonase-1 may reflect a pro-oxidant state in specific subgroups of patients with schizophrenia that require further assessment in appropriately designed studies.

## 1. Introduction

There is increasing evidence from experimental models and human studies that schizophrenia and associated psychiatric disorders, e.g., schizoaffective disorder, are characterised by a dysregulated immune system and a pro-oxidative state [1,2,3,4,5]. Local inflammation (neuroinflammation) and abnormalities in the balance between antioxidant and pro-oxidant mechanisms are likely to be involved in the pathogenesis and the progression of schizophrenia [6,7,8,9]. However, the co-existence of a systemic pro-inflammatory and pro-oxidant state may also account for the increased risk of several other disease states in patients with schizophrenia, which imposes a further health and financial burden to this patient group as well as to healthcare systems worldwide [10,11,12,13]. For example, several epidemiological studies have recently reported a significant increase in the risk of atherosclerosis and cardiovascular disease, conditions that are also characterised by a pro-inflammatory and pro-oxidant state, in patients with schizophrenia [14,15,16,17,18]. These observations support the proposition that oxidative stress plays a critical pathophysiological role in the onset and the progression of schizophrenia and in the association between schizophrenia and atherosclerosis and cardiovascular disease. Furthermore, they suggest that the identification of circulating biomarkers of oxidative stress may be useful for risk stratification as well as for the monitoring of the clinical effects of specific antipsychotic and cardioprotective treatments in patients with schizophrenia [1,19,20,21,22,23,24,25]. One such potential biomarker is paraoxonase-1, an antioxidant calcium-dependent high-density lipoprotein (HDL)-associated glycoprotein esterase containing 354 amino acids which possesses a combined paraoxonase and arylesterase activity [26]. The hydrophobic sequence located in the N-terminal region of paraoxonase-1 favors its interaction with HDL [27,28].

The paraoxonase multi-gene family is widely expressed in mammals and includes paraoxonase-1, paraoxonase-2, and paraoxonase-3 [26]. Paraoxonase-1 and paraoxonase-3 are mainly present in the circulation whereas paraoxonase-2 is located intracellularly [29]. Paraoxonase-1 is the most studied in basic research and clinical studies as it has a wider range of substrates and circulating concentrations that are at least twofold those of paraoxonase-3 [30,31]. Consequently, the assessment of circulating paraoxonase-1 likely reflects the majority of circulating paraoxonase activity. This includes paraoxonase activity (which uses the substrate paraoxon) and arylesterase activity (which uses the substrate phenylacetate) [31,32,33,34]. A number of studies have recently highlighted the potential clinical utility of measuring the circulating concentrations of paraoxonase-1 in several disease states, particularly in autoimmune, circulatory, and respiratory disorders [35,36,37,38,39,40,41]. Paraoxonase-1 exerts a critical role in maintaining the antioxidant effects of HDL, and its reduction is believed to promote a dysfunctional HDL which, in turn, can paradoxically favor the onset of atherosclerosis and other inflammatory conditions [28,42,43,44,45,46]. For example, in a meta-analysis of six observational studies including a total of 15,064 subjects, the pooled age-adjusted risk ratio (RR) for any cardiovascular event per each one standard deviation (SD) increase in paraoxonase-1 concentration was 0.87 (95% confidence intervals, CI, 0.80 to 0.96, *p* = 0.005) [47]. Therefore, a reduction in paraoxonase-1 concentration might account for a systemic pro-oxidative state as well as the increased cardiovascular risk observed in patients with schizophrenia.

We investigated this issue by conducting a systematic review and meta-analysis of studies reporting circulating paraoxonase-1 concentrations in patients with schizophrenia and healthy controls and assessing possible associations between the effect size of paraoxonase and arylesterase activity and pre-defined variables. We speculated that schizophrenia is associated with a significant reduction in the enzymatic activity of paraoxonase and arylesterase, reflecting the presence of a pro-oxidant state in this patient group.

## 2. Methods

### 2.1. Literature Search

We searched PubMed, Web of Science, and Scopus from inception to 31 May 2023. The following terms and their combination were used in the systematic search: “paraoxonase” or “PON” or “arylesterase” and “schizophrenia” or “acute psychotic disorder” or “schizoaffective disorder”. Two independent investigators screened the abstracts and, if relevant, individual articles according to the following criteria: (i) the assessment of plasma or serum paraoxonase-1 concentrations; (ii) the assessment of paraoxonase and/or arylesterase activity in patients with schizophrenia and in healthy controls (case-control design); (iii) the assessment of 10 or more patients; (iv) the use of English language; and (v) the availability of the full-text of the article. The 2 investigators also searched the references of individual articles for additional studies. A third investigator was involved in case of disagreement.

Extracted parameters included: first author, year of publication, study country, number of participants, age, sex distribution, body mass index, serum concentrations of paraoxonase, arylesterase, and HDL–cholesterol, and pharmacological treatment for schizophrenia. We assessed the risk of bias (Joanna Briggs Institute critical appraisal checklist for analytical studies; studies addressing ≥ 75% checklist items were adjudicated as low risk [48]) and the certainty of evidence (grades of recommendation, assessment, development and evaluation, GRADE, working group system [49,50]). We fully complied with the preferred reporting items for systematic reviews and meta-analyses (PRISMA) 2020 statement (Supplementary Tables S1 and S2) [51] and registered our review in the International Prospective Register of Systematic Reviews (PROSPERO registration number: CRD42023435442).

### 2.2. Statistical Analysis

Forest plots of standardized mean differences (SMD) and 95% confidence intervals (CIs) were generated to evaluate the presence of differences in paraoxonase-1 concentrations between patients with schizophrenia and healthy controls (statistical significance set at *p* < 0.05). Means and standard deviations were extrapolated from medians and interquartile ranges or ranges [52,53], or using the Graph Data Extractor software (San Diego, CA, USA). The presence of between-study SMD heterogeneity was assessed using the Q statistic (statistical significance set at *p* < 0.10) and the I^2^ statistic. [54,55,56]. The stability of the effect size was assessed in a sensitivity analysis [57]. Publication bias was assessed with the Begg’s and Egger’s tests [58,59] and the “trim-and-fill” method [60]. Univariate meta-regression and subgroup analyses were conducted to investigate the presence of associations between the effect size and several pre-defined variables: age, proportion of males, year of publication, study country, sample size, body mass index, HDL–cholesterol concentrations, and pharmacological treatment for schizophrenia. Statistical analyses were performed using Stata 14 (STATA Corp, College Station, TX, USA).

## 3. Results

### 3.1. Systematic Search

**Figure 1:**
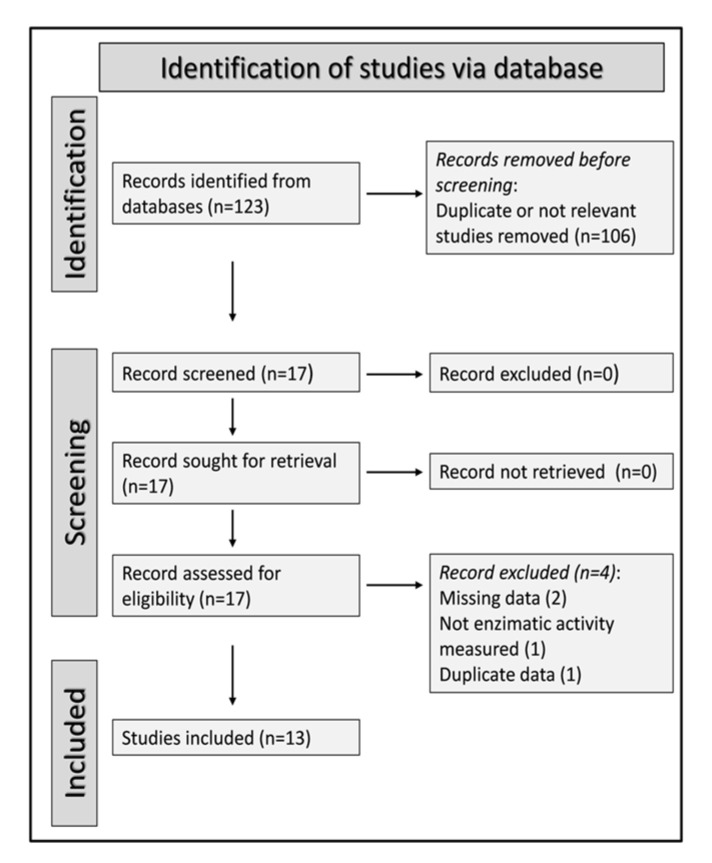
PRISMA 2020 flow diagram.

A flow chart of the screening process is described in Figure 1. After identifying a total of 123 articles, 106 were excluded as they were either duplicates or not relevant to the search strategy. After a full-text revision of the remaining 17 articles, 4 were further excluded (missing information: 2 studies; duplicate data: 1 study; paraoxonase-1 assay not performed as enzyme activity: 1 study), leaving 13 studies for final analysis [61,62,63,64,65,66,67,68,69,70,71,72,73]. The selected studies, published between 2007 and 2018, investigated a total of 825 patients with schizophrenia with a mean age of 36 years (66% males) and 725 healthy controls with a mean age of 36 years (58% males; Table 1).

### 3.2. Paraoxonase Activity

Nine studies that included 13 study groups investigated paraoxonase activity in a total of 570 patients with schizophrenia (mean age 37 years, 65% males) and 512 healthy controls (mean age 38 years, 59% males) [61,62,63,64,65,68,69,70,71]. Seven studies were conducted in Turkey [61,62,63,68,69,70,71], one in Romania [64], and one in Tunisia [65]. Patients received pharmacological treatment for schizophrenia in 11 study groups [61,62,63,64,65,68,69,70,71] and no treatment in the remaining 2 [61,68]. The enzymatic activity of paraoxonase was evaluated by using paraoxon as a substrate in all the selected studies [61,62,63,64,65,68,69,70,71]. Furthermore, all studies had a low risk of bias (Supplementary Table S3) [61,62,63,64,65,68,69,70,71].

**Figure 2:**
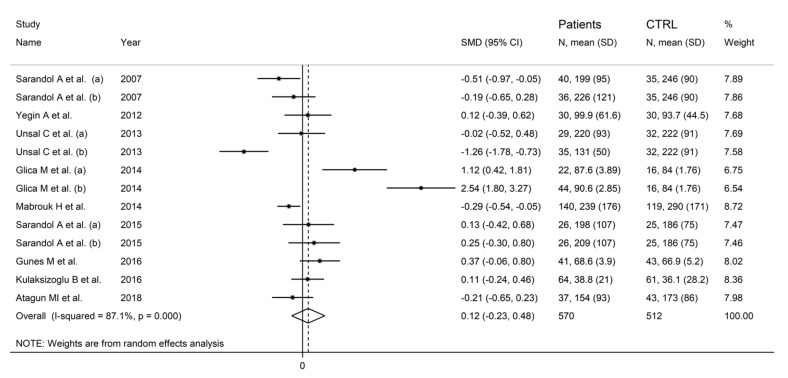
Paraoxonase activity in patients with schizophrenia and healthy controls. (a) and (b) refer to different arms of the same study [61,62,63,64,65,68,69,70,71].

**Figure 3:**
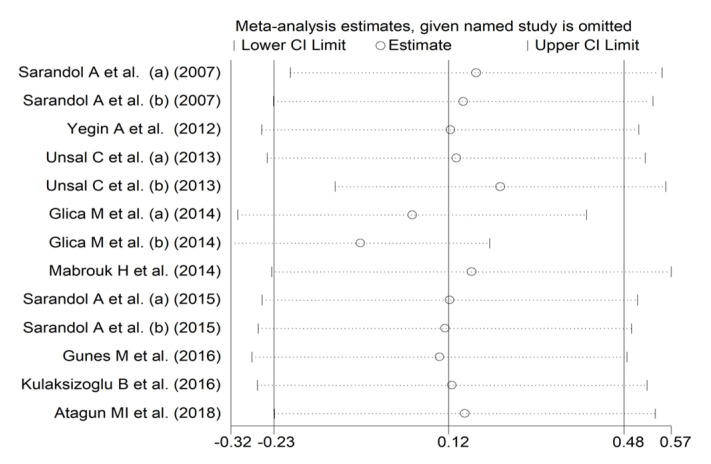
Sensitivity analysis of the association between paraoxonase activity and schizophrenia. (a) and (b) refer to different arms of the same study [61,62,63,64,65,68,69,70,71].

In forest plots, paraoxonase activity was non-significantly different between control subjects and patients with schizophrenia (SMD = 0.12, 95% CI −0.23 to 0.48, *p* = 0.50; I^2^ = 87.1%, *p* < 0.001; Figure 2). A sensitivity analysis showed stability of the effect size, with the corresponding SMD values ranging between −0.06 and 0.13 (Figure 3).

**Figure 4:**
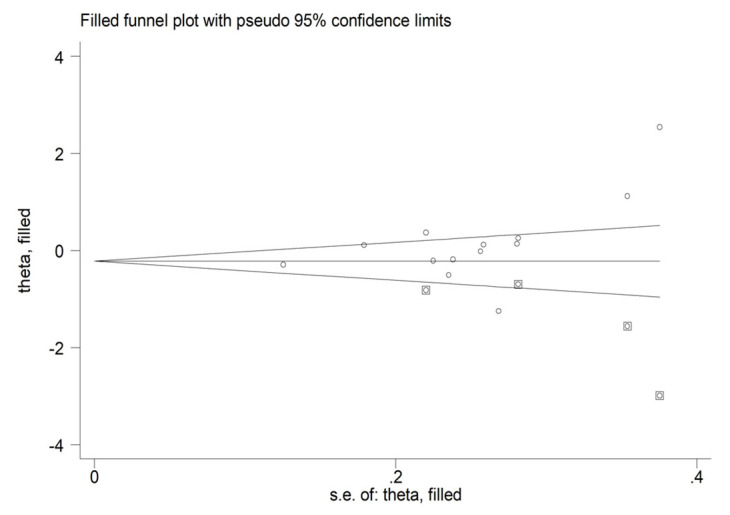
Funnel plot of paraoxonase activity and schizophrenia after “trimming-and-filling”.

There was a significant publication bias with the Begg’s test (*p* = 0.04) but not with the Egger’s test (*p* = 0.10). The “trim-and-fill” procedure identified four studies to be added to the funnel plot (Figure 4). The resulting SMD was −0.23 (95% CI −0.62 to 0.16, *p* = 0.25).

**Figure 5:**
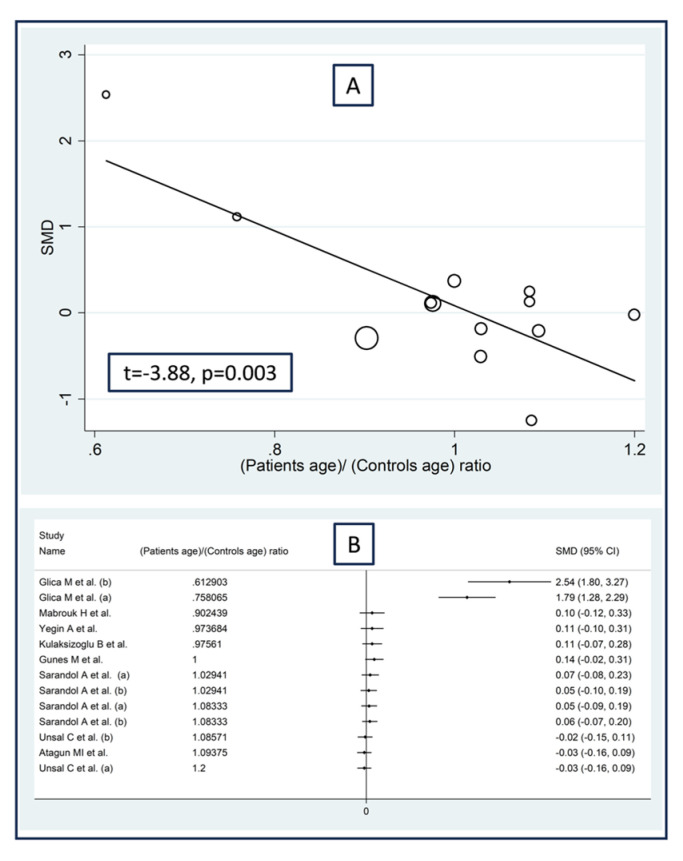
Association between the effect size of paraoxonase activity and patient/control age ratio (**A**), and cumulative meta-analysis of paraoxonase activity based on patient/control age ratio (**B**). (a) and (b) refer to different arms of the same study [61,62,63,64,65,68,69,70,71].

**Figure 6:**
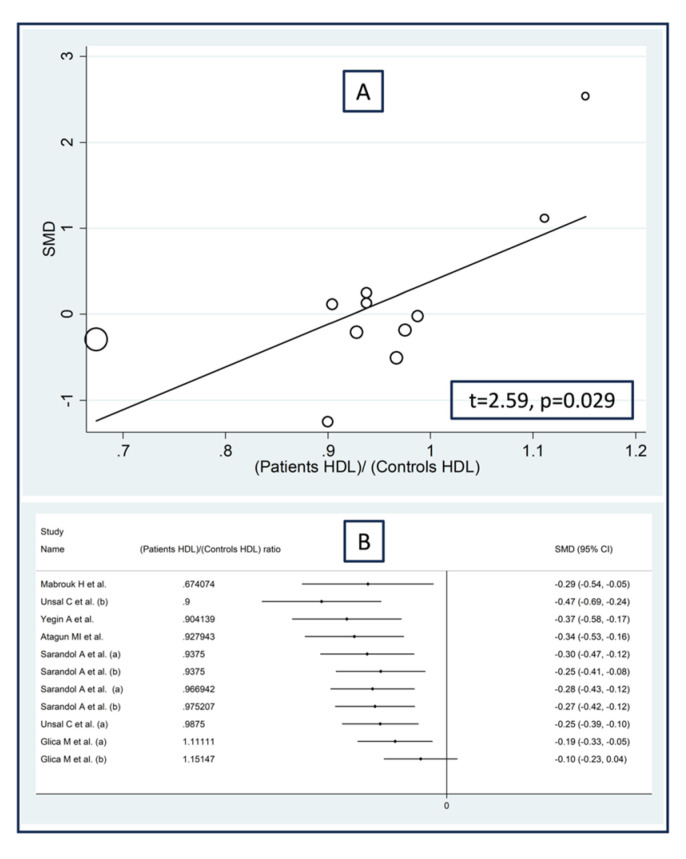
Association between the effect size of paraoxonase activity and patient/control HDL–cholesterol ratio (**A**), and cumulative meta-analysis of paraoxonase activity based on patient/control HDL–cholesterol ratio (**B**). (a) and (b) refer to different arms of the same study [61,62,63,64,65,68,71].

Non-significant associations were observed between the effect size of paraoxonase activity and the proportion of males (t = −1.68, *p* = 0.13), the publication year (t = 0.76, *p* = 0.46), the sample size (t = −0.79, *p* = 0.45), or the body mass index (t = 2.24, *p* = 0.052) in the meta-regression analysis. By contrast, there was a significant negative association with the patient/control age ratio (t = −3.88, *p* = 0.003; Figure 5A) and a significant positive association between the effect size and the patient/control HDL–cholesterol ratio (t = 2.59, *p* = 0.029; Figure 6A). These associations were also confirmed using the metacum analysis command (Figure 5B and Figure 6B).

**Figure 7:**
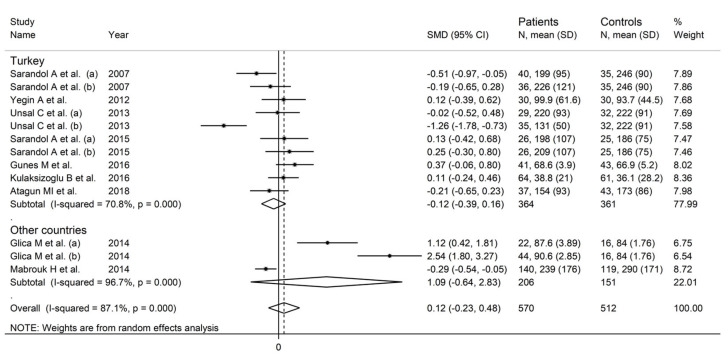
Paraoxonase activity in schizophrenic patients and healthy controls according to study country. (a) and (b) refer to different arms of the same study [61,62,63,64,65,68,69,70,71].

**Figure 8:**
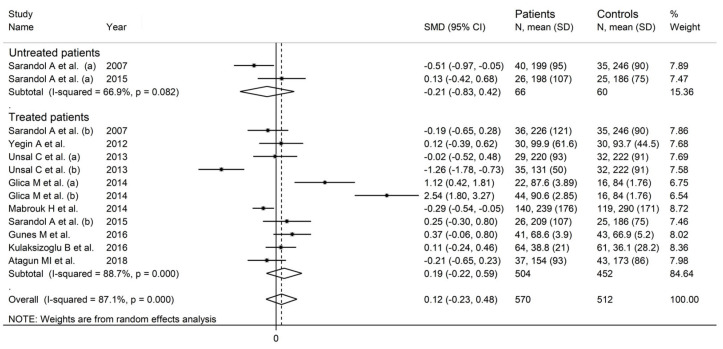
Paraoxonase activity in schizophrenic patients and healthy controls according to pharmacological treatment of schizophrenia. (a) and (b) refer to different arms of the same study [61,62,63,64,65,68,69,70,71].

In a subgroup analysis, the SMD was significantly smaller (*p* = 0.04) in studies conducted in Turkey (SMD = −0.12, 95% CI −0.39 to 0.16, *p* = 0.41; I^2^ = 70.8%, *p* < 0.001) than those conducted in other countries (SMD = 1.09, 95% CI −0.64 to 2.83, *p* = 0.22; I^2^ = 96.7%, *p* < 0.001; Figure 7). There were non-significant differences (*p* = 0.58) in the pooled SMD between studies performed in untreated patients (SMD = −0.21, 95% CI −0.83 to 0.42, *p* = 0.51; I^2^ = 66.9%, *p* = 0.082) and those in treated patients (SMD = 0.19, 95% CI −0.22 to 0.59, *p* = 0.37; I^2^ = 88.7%, *p* < 0.001; Figure 8).

The low level of certainty for cross-sectional studies (rating 2, ⊕⊕⊝⊝) was downgraded to extremely low (rating 0, ⊝⊝⊝⊝) after considering the high and unexplained heterogeneity, the relatively high imprecision, and the presence of publication bias.

### 3.3. Arylesterase Activity

Ten studies including 14 study groups, all with a low risk of bias (Supplementary Table S3), reported arylesterase activity in a total of 562 patients (mean age 34 years, 65% males) and 507 healthy controls (mean age 35 years, 59% males) [61,62,64,66,67,68,69,71,72,73]. Five studies were conducted in Turkey [61,62,68,69,71], two in Romania [64,73], two in Brazil [66,72], and one in Israel [67]. Ten study groups investigated patients receiving pharmacological treatment for schizophrenia [61,62,64,67,68,69,71,72,73] and four untreated patients [61,66,67,68]. Arylesterase activity was evaluated by using phenylacetate as a substrate in all studies [61,62,64,66,67,68,69,71,72,73].

**Figure 9:**
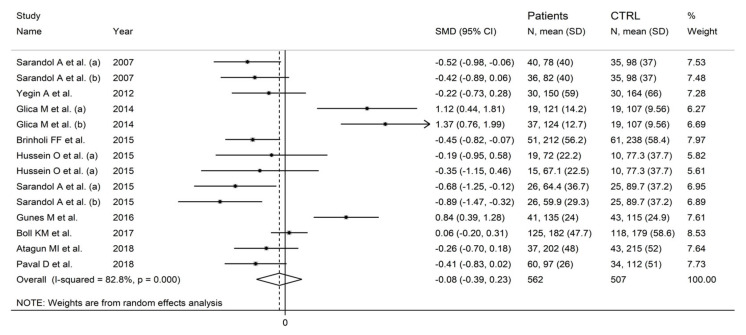
Arylesterase activity in patients with schizophrenia and healthy controls. (a) and (b) refer to different arms of the same study [61,62,64,66,67,68,69,71,72,73].

**Figure 10:**
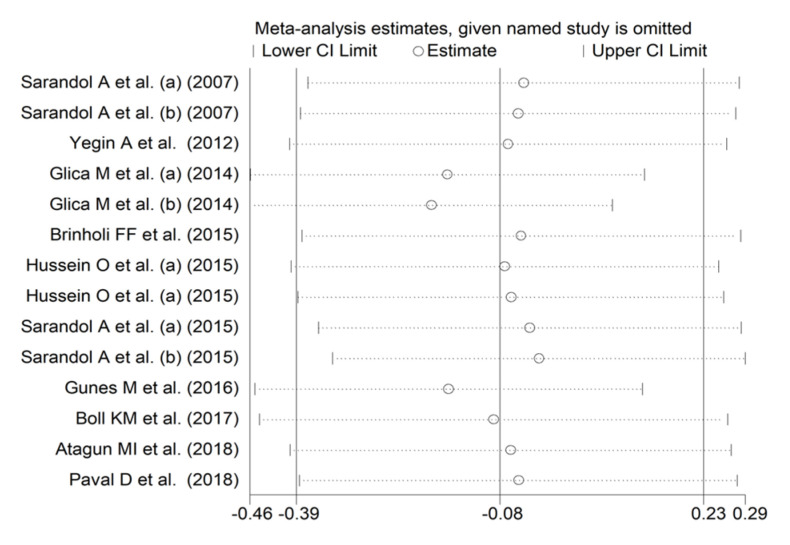
Sensitivity analysis of the association between arylesterase activity and schizophrenia. (a) and (b) refer to different arms of the same study [61,62,64,66,67,68,69,71,72,73].

In forest plots, arylesterase activity was non-significantly different between control subjects and patients with schizophrenia (SMD = −0.08, 95% CI −0.39 to 0.23; *p* = 0.61; I^2^ = 82.8%, *p* < 0.001; Figure 9). A sensitivity analysis showed stability of the results, with pooled SMD values ranging between −0.16 and −0.02; Figure 10).

**Figure 11:**
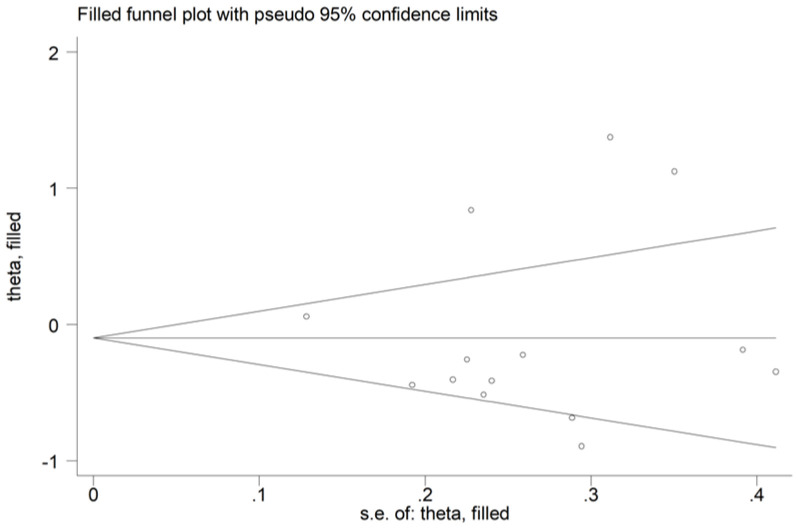
Funnel plot of the association between arylesterase activity and schizophrenia after “trimming-and-filling”.

There was no publication bias with the Begg’s test (*p* = 0.66), the Egger’s test (*p* = 0.75), or the “trim-and-fill” method (Figure 11).

**Figure 12:**
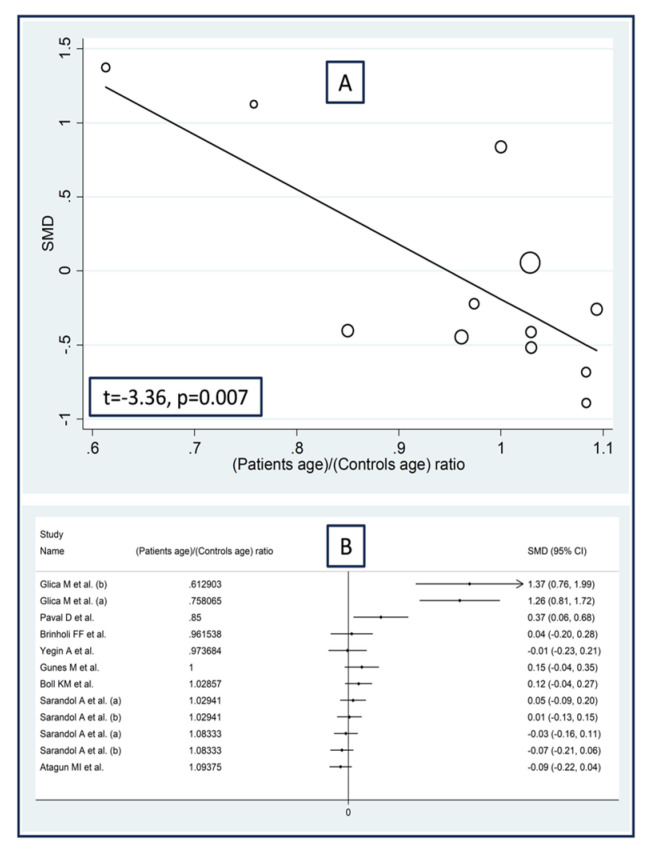
Association between the effect size of arylesterase activity and patient/control age ratio (**A**), and cumulative meta-analysis of arylesterase activity based on patient/control age ratio (**B**). (a) and (b) refer to different arms of the same study [61,62,64,66,68,69,71,72,73].

**Figure 13:**
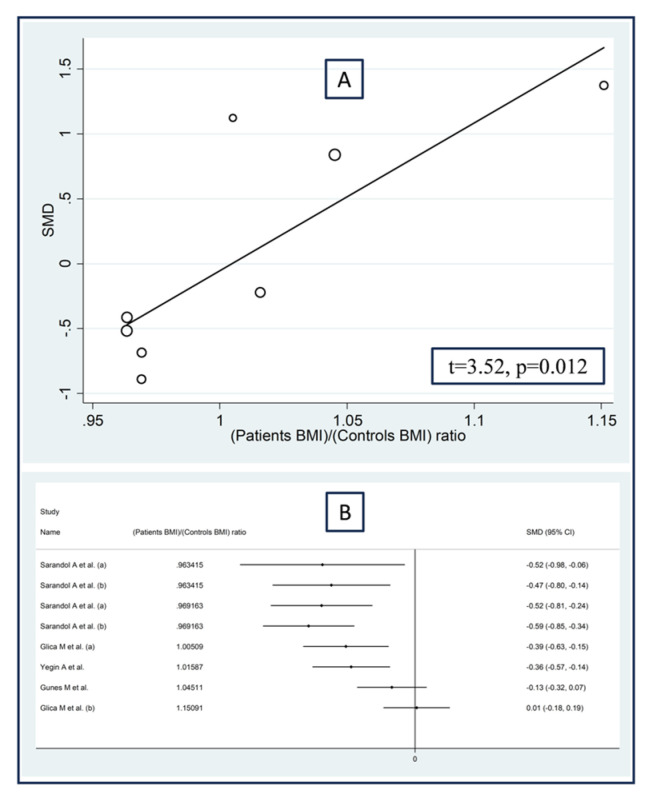
Association between the effect size of arylesterase activity and patient/control body mass index ratio (**A**), and cumulative meta-analysis of arylesterase activity based on patient/control body mass index ratio (**B**). (a) and (b) refer to different arms of the same study [61,62,64,68,69].

**Figure 14:**
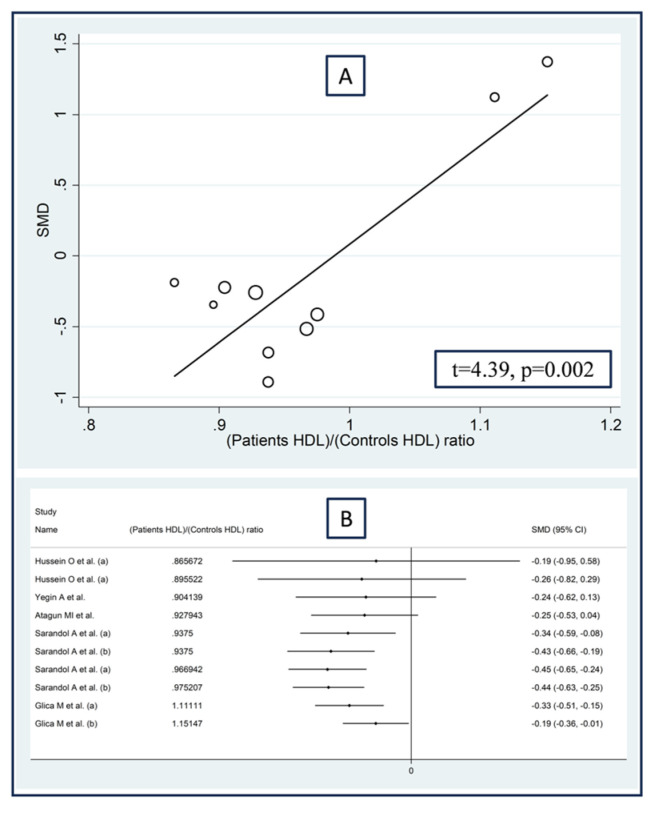
Association between the effect size of arylesterase activity and patient/control HDL–cholesterol ratio (**A**), and cumulative meta-analysis of arylesterase activity based on patient/control HDL–cholesterol ratio (**B**). (a) and (b) refer to different arms of the same study [61,62,64,67,68,71].

No significant associations were observed in meta-regression between the effect size and the proportion of males (t = −1.07, *p* = 0.31), the publication year (t = 0.46, *p* = 0.65), or the sample size (t = −0.02, *p* = 0.99). By contrast, we observed significant positive associations between the effect size and the patient/control age ratio (t = −3.36, *p* = 0.007; Figure 12A), the patient/control body mass index ratio (t = 3.52, *p* = 0.012; Figure 13A), and the patient/control HDL–cholesterol ratio (t = 4.39, *p* = 0.002; Figure 14A). Similar results were observed using the metacum analysis command (Figure 12B, Figure 13B and Figure 14B).

**Figure 15:**
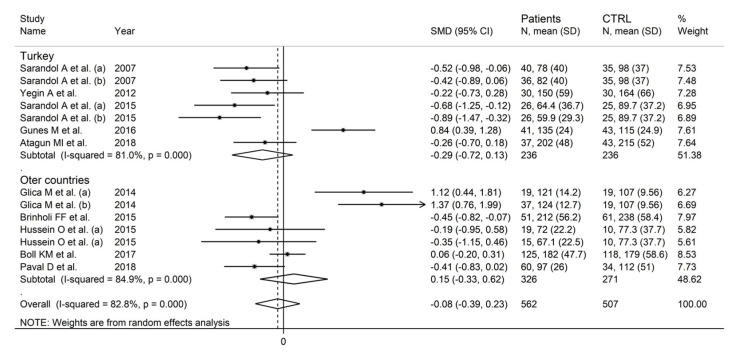
Arylesterase activity in schizophrenic patients and healthy controls according to study country. (a) and (b) refer to different arms of the same study [61,62,64,66,67,68,69,71,72,73].

**Figure 16:**
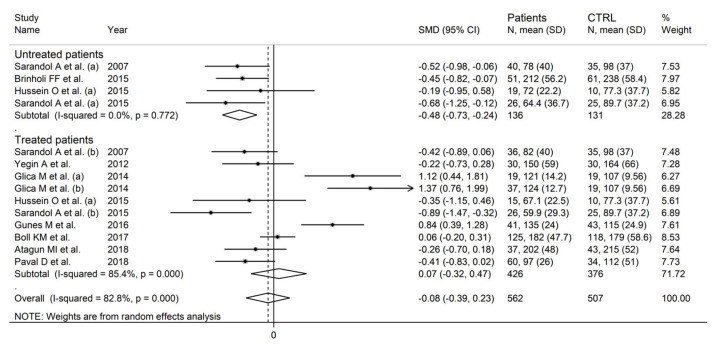
Arylesterase activity in schizophrenic patients and healthy controls according to pharmacological treatment of schizophrenia. (a) and (b) refer to different arms of the same study [61,62,64,66,67,68,69,71,72,73].

In the subgroup analysis, there were non-significant differences (*p* = 0.23) in SMD values between studies conducted in Turkey (SMD = −0.29, 95% CI −0.72 to 0.13, *p* = 0.18; I^2^ = 81.0%, *p* < 0.001) and those conducted in other countries (SMD = 0.15, 95% CI −0.33 to 0.62, *p* = 0.54; I^2^ = 84.9%, *p* < 0.001; Figure 15). By contrast, the pooled SMD was significantly lower in studies conducted in untreated patients (SMD = −0.48, 95% CI −0.73 to −0.24, *p* < 0.001; I^2^ = 0.0%, *p* = 0.77) but not in those conducted in treated patients (SMD = 0.07, 95% CI −0.32 to 0.47, *p* = 0.71; I^2^ = 85.4%, *p* < 0.001; Figure 16), with a virtually absent heterogeneity in the former subgroup.

The low level of certainty for cross-sectional studies (rating 2, ⊕⊕⊝⊝) was downgraded to very low (rating 1, ⊕⊝⊝⊝) after taking into account the relatively high imprecision (relatively large confidence intervals).

## 4. Discussion

In our systematic review and meta-analysis, the enzymatic activity of paraoxonase and arylesterase, two essential components of circulating paraoxonase-1, were non-significantly different between patients with schizophrenia and healthy controls. However, in a meta-regression analysis we identified the presence of significant associations between the effect size of paraoxonase activity and patient/control age and HDL–cholesterol ratios, and between the effect size of arylesterase activity and patient/control age, body mass index, and HDL–cholesterol ratios. Furthermore, in a subgroup analysis the effect size of paraoxonase activity was significantly associated with the study country, whereas the effect size of arylesterase was significantly associated with pharmacological treatment for schizophrenia. In sensitivity analyses, the pooled SMD values of paraoxonase and arylesterase activity were not substantially influenced by individual studies, suggesting stability of the results of the meta-analysis.

The increasing evidence of a state of oxidative stress associated with schizophrenia has stimulated a significant body of research investigating the clinical significance of alterations in the redox state in this condition as well as other associated co-morbidities. In this context, studies have recently investigated the association between several biomarkers of oxidative stress, the duration of schizophrenia, and specific symptoms and psychopathological features of the disease, with contrasting results in terms of the direction of the observed associations [9,74]. Pending the results of additional investigations seeking to address this issue, the observed inconsistency in the available evidence could be related, at least in part, to the multiple biochemical and molecular pathways involved in redox homeostatic mechanisms and the complex analytical procedures required for the determination of biomarkers of oxidative stress in the blood and other biological matrices, with the potential for error [75,76]. Another factor that is likely to play an important role is represented by the interindividual variability in the clinical and demographic characteristics of the study participants [9].

In terms of inter-individual variability in patient characteristics, there is good evidence that the redox state can be significantly influenced by age [77], gender [78], and several other sociodemographic and biochemical factors, particularly cigarette smoking, C-reactive protein concentrations, hyperglycaemia, nutrients, physical activity, and body mass index [79,80]. This complexity could explain, at least in part, some of the results of our meta-regression and subgroup analyses, particularly the significant negative associations between the SMD of paraoxonase/arylesterase activity and the patient/control age ratio and the significant positive associations between the SMD of paraoxonase/arylesterase and the patient/control HDL–cholesterol ratio. In other words, our analyses suggest that further studies are more likely to observe significant reductions in paraoxonase/arylesterase activity in subgroups of schizophrenic patients with older age and low HDL–cholesterol concentrations rather than older patients with high HDL–cholesterol concentrations and younger patients with low or high HDL–cholesterol concentrations. This hypothesis is also in line with the reported associations between schizophrenia and other conditions characterized by a pro-oxidative state, particularly atherosclerosis and cardiovascular disease, that are also more likely to affect older schizophrenic patients with relatively low HDL–cholesterol concentrations, given the established role of advancing age and low HDL–cholesterol as cardiovascular risk factors [81,82].

The presence of a strong association between schizophrenia and atherosclerotic cardiovascular disease has been highlighted in a systematic review and meta-analysis that included 38 studies, of which 29 were longitudinal, assessing a total of 1,591,106 patients with schizophrenia. In cross-sectional studies, schizophrenia was significantly associated with the presence of coronary heart disease (adjusted odds ratio, OR = 1.52, 95% CI 1.48 to 1.56, *p* < 0.001), cerebrovascular disease (adjusted OR = 2.05, 95% CI 1.59 to 2.64, *p* < 0.001), and congestive heart failure (adjusted OR = 1.60, 95% CI 1.06 to 2.40, *p* = 0.02). Similar results were observed in longitudinal studies where schizophrenia was significantly associated with incident cardiovascular disease (adjusted hazard ratio, HR = 1.95, 95% CI 1.41 to 2.70, *p* < 0.001), incident coronary heart disease (adjusted HR = 1.59, 95% CI 1.08 to 2.35, *p* = 0.02), incident cerebrovascular disease (adjusted HR = 1.57, 1.09 to 2.25, *p* = 0.02), and incident congestive heart failure (unadjusted relative risk, RR = 1.80, 95% CI 1.15 to 2.79, *p* = 0.009) [14]. A number of mechanisms have been suggested to account for the increased cardiovascular risk in patients with schizophrenia, including the high prevalence of smoking, unhealthy dietary patterns, and physical inactivity [17,83]. Studies have also reported a significant reduction in the concentrations of HDL–cholesterol, a cholesterol fraction that is critically linked with paraoxonase-1, in patients with schizophrenia [84,85,86]. In one study, patients with acute-phase schizophrenia had significantly lower HDL concentrations compared to healthy subjects (43 ± 14 vs. 53 ± 13 mg/dL, *p* = 0.03). Interestingly, a significant increase in HDL–cholesterol was observed in patients who responded to three-week treatment with atypical antipsychotic drugs (+4 ± 8 mg/dL, *p* = 0.007) but not with conventional antipsychotics (+0.5 ± 14 mg/dL, *p* = 0.82) [87]. The beneficial effects of some antipsychotic agents on HDL–cholesterol concentrations have also been reported in a systematic review and meta-analysis of 22 studies that investigated the effects of 10 different antipsychotics in 7073 patients compared to placebos in 2189 patients. Aripiprazole and brexpiprazole were the antipsychotic agents causing the greatest increase in HDL–cholesterol concentrations vs. placebo [88]. Additional studies should investigate the presence of possible associations between paraoxonase/arylesterase activity, individual cardiovascular risk factors, and clinical cardiovascular endpoints (e.g., myocardial infarction, stroke, and congestive heart failure) in schizophrenia. These studies should also determine whether such associations are influenced by age, sex, ethnicity, specific disease subtypes, HDL–cholesterol concentrations, and treatment with specific antipsychotic drugs.

Another interesting observation in our subgroup analysis was the presence of significant reductions in arylesterase activity in studies of untreated patients but not in those of patients receiving pharmacological treatment for schizophrenia. Albeit hypothesis-generating, this finding suggests that treatment with antipsychotic drugs might increase arylesterase activity to levels that are comparable to those observed in healthy subjects. This would dilute the differences in arylesterase activity between treated patients and healthy controls. A systematic review and meta-analysis published in 2011 did not report any conclusive evidence regarding the effect of antipsychotic drugs on oxidative stress [89]. However, a more recent systematic review has reported that olanzapine, paliperidone, risperidone, and haloperidol prevent oxidative stress-induced neurotoxicity in in vitro studies [90]. Clearly, more research is warranted to investigate the effect of individual antipsychotic drugs on oxidative stress in patients with schizophrenia in studies that also include a range of genetic polymorphisms of paraoxonase as a potential modulating factor [91,92,93,94]. This issue is particularly relevant as there is good evidence that treatment with antipsychotic medications, particularly atypical antipsychotics, is also associated with weight gain, insulin resistance, diabetes, and dyslipidaemia in this patient group, with a consequent increase in the overall cardiovascular risk [95,96,97]. At the same time, however, a large observational study in 62,250 patients with schizophrenia followed for 20 years has reported that antipsychotic treatment is associated with reduced all-cause mortality and cardiovascular mortality in schizophrenia, with an adjusted HR of 0.62 (95% CI 0.57 to 0.67) for cardiovascular mortality. Notably, even in the presence of significant variability, virtually all studied antipsychotic agents were associated with a reduced risk of cardiovascular mortality in this study, particularly quetiapine (HR = 0.51, 95% CI 0.43 to 0.61), aripiprazole (HR = 0.52, 95% CI 0.30 to 0.89), clozapine (HR = 0.55, 95% CI 0.46 to 0.64), and risperidone (HR = 0.64, 95% CI 0.56 to 0.73) [98]. In another study, the duration of treatment with antipsychotics (including typical and atypical antipsychotics) was significantly associated with a reduced risk of cardiovascular events in patients with schizophrenia (adjusted OR = 0.95, 95% CI 0.91 to 0.99, *p* = 0.014) after adjusting for age, urea, creatinine, uric acid, thyroxine, leukocytes, body mass index, and diastolic blood pressure [99]. Large, well-designed prospective studies are likely to shed light on the complex interplay between schizophrenia, oxidative stress assessed by measuring paraoxonase/arylesterase activity, antipsychotic drugs, and cardiovascular disease.

Strengths of our study are the combined assessment of paraoxonase and arylesterase enzymatic activity, essential components of paraoxonase-1, the comprehensive set of meta-regression and subgroup analyses, the rigorous assessment of the risk of bias and the certainty of evidence, and the stability of the results after sequentially removing individual studies in a sensitivity analysis. A significant limitation is that a significant proportion of studies was conducted in one country, Turkey, which limits the generalisability of the results to other patient populations, particularly European, North American, and Eastern Asian patients. This issue requires further research as studies conducted in USA and South Africa have reported the presence of significant ethnic-related differences in paraoxonase-1 [100,101,102].

## 5. Conclusions

Our study has shown the presence of non-significant differences in circulating paraoxonase-1, expressed as paraoxonase and arylesterase enzyme activity, between patients with schizophrenia and healthy controls. However, the results of meta-regression and subgroup analyses suggest that such differences may be significant in subgroups of patients with schizophrenia, particularly older patients with relatively low HDL–cholesterol concentrations. Further studies should address this issue by investigating paraoxonase-1 and the possible effects of antipsychotic drugs and other antioxidant strategies on disease progression and cardiovascular risk in these subgroups and consider the modulating effect of genetic polymorphisms of paraoxonase-1 and ethnic differences.

## Figures and Tables

**Table 1:** Characteristics of the selected studies.

	Controls						Patients with Schizophrenia					
Study	n	Age (Years)	M/F	PON Mean ± SD (U/L)	ARE Mean ± SD (kU/L)	HDL Mean (mg/dL or mmol/L)	n	Age (Years)	M/F	PON Mean ± SD (U/L)	ARE Mean ± SD (kU/L)	HDL Mean (mg/dL or mmol/L)
Sarandol A. et al. (a), 2007, Turkey [61]	35	34	17/18	246 ± 90	98 ± 37	1.2	40	35	18/22	199 ± 95	78 ± 40	1.2
Sarandol A. et al. (b) 2007, Turkey [61]	35	34	NR	246 ± 90	98 ± 37	1.2	36	35	NR	226 ± 121	82 ± 40	1.2
Yegin A. et al., 2012, Turkey [62]	30	38	30/0	93.7 ± 44.5	164 ± 66	45.9	30	37	30/0	99.9 ± 61.6	150 ± 59	41.5
Unsal C. et al. (a), 2013, Turkey [63]	32	35	21/11	222 ± 91	NR	40	29	42	14/15	220 ± 93	NR	39.5
Unsal C. et al. (b), 2013, Turkey [63]	32	35	21/11	222 ± 91	NR	40	35	38	15/20	131 ± 50	NR	36
Gilca M. et al. (a), 2014, Romania [64]	16	62	NR	84.0 ± 1.8	107.4 ± 9.6	37.2	22	47	NR	87.5 ± 3.9	121.0 ± 14.2	41.3
Gilca M. et al. (b), 2014, Romania [64]	16	62	NR	84.0 ± 1.8	107.4 ± 9.6	37.2	44	38	NR	90.6 ± 2.8	123.6 ± 12.7	42.8
Mabrouk H. et al., 2014, Tunisia [65]	119	41	64/55	290 ± 171	NR	1.35	140	37	116/24	239 ± 176	NR	0.91
Brinholi F.F. et al., 2015, Brazil [66]	61	26	32/29	NR	237.8 ± 58.4	NR	51	25	36/15	NR	212.2 ± 56.2	NR
Hussein O. et al. (a), 2015, Israel [67]	10	NR	13/6	NR	77.3 ± 37.7	1.34	19	35	4/6	NR	72 ± 22.2	1.2
Hussein O. et al. (a), 2015, Israel [67]	10	NR	NR	NR	77.3 ± 37.7	1.34	15	NR	NR	NR	67.1 ± 22.5	1.2
Sarandol A. et al. (a), 2015, Turkey [68]	25	24	10/15	186 ± 75	89.7 ± 37.2	48	26	26	10/16	198 ± 107	64.4 ± 36.7	45
Sarandol A. et al. (b), 2015, Turkey [68]	25	24	10/15	186 ± 75	89.7 ± 37.2	48	26	26	10/16	209 ± 107	59.9 ± 29.3	45
Gunes M. et al., 2016, Turkey [69]	43	35	36/7	66.9 ± 5.2	114.5 ± 24.9	NR	41	35	35/6	68.6 ± 3.9	135 ± 24	NR
Kulaksizoglu B. et al., 2016, Turkey [70]	61	41	33/28	36.1 ± 28.2	NR	NR	64	40	36/28	38.7 ± 21.0	NR	NR
Boll K.M. et al., 2017, Brazil [72]	118	35	74/44	NR	178.6 ± 58.6	NR	125	36	85/40	NR	181.6 ± 47.7	NR
Atagun M.I. et al., 2018, Turkey [71]	43	32	20/23	186 ± 75	215 ± 52	46.63	37	35	19/18	154 ± 93	202 ± 48	43.27
Paval D. et al., 2018, Romania [73]	34	40	12/22	NR	112 ± 51	1.21	60	34	42/18	NR	97 ± 26	1.17

Legend: PON, paraoxonase-1; ARE, arylesterase; HDL, high-density lipoprotein cholesterol; M, male; F, female; NR, not reported. (a) and (b) refer to different arms of the same study.

## Data Availability

The relevant data are available from A.Z. upon reasonable request.

## Supplementary Materials

The following supporting information can be downloaded at: https://www.mdpi.com/article/10.3390/antiox12081484/s1, Table S1: PRISMA 2020 for abstracts checklist; Table S2: PRISMA 2020 checklist; Table S3: The Joanna Briggs Institute critical appraisal checklist.
