# Supplementary material for: Paraoxonase/Arylesterase Activity of Serum Paraoxonase-1 and Schizophrenia: A Systematic Review and Meta-Analysis
Source: Antioxidants (Basel). 2023 Jul 25;12(8):1484. doi: 10.3390/antiox12081484 (PMC10451270; doi:10.3390/antiox12081484)
Supplement: Supplementary file 1 [file Supplementary_Table_3.pdf › Supplementary_Table_3.pdf]

Supplementary Table 3. The Joanna Briggs Institute critical appraisal checklist.

Study	Were the criteria for inclusion clearly defined?	Were the subjects and the setting described in detail?	Was the exposure measured in a valid and reliable way?	Were objective, standard criteria used for measurement of the condition?	Were confounding factors identified?	Were strategies to deal with confounding factors stated?	Were the outcomes measured in a valid and reliable way?	Was appropriate statistical analysis used?	Risk of bias
Sarandol A et al, 2007	Yes	Yes	Yes	Yes	No	No	Yes	Yes	Low
Yegin A et al, 2012	Yes	Yes	Yes	Yes	No	No	Yes	Yes	Low
Unsal C et al, 2013	Yes	Yes	Yes	Yes	No	No	Yes	Yes	Low
Gilca M et al, 2014	Yes	Yes	Yes	Yes	No	No	Yes	Yes	Low
Mabrouk H et al, 2014	Yes	Yes	Yes	Yes	No	No	Yes	Yes	Low
Brinholi FF et al, 2015	Yes	Yes	Yes	Yes	Yes	Yes	Yes	Yes	Low
Hussein O et al, 2015	Yes	Yes	Yes	Yes	No	No	Yes	Yes	Yes
Sarandol A et al, 2015	Yes	Yes	Yes	Yes	No	No	Yes	Yes	Low
Gunes M et al, 2016	Yes	Yes	Yes	Yes	No	No	Yes	Yes	Low
Kulaksizoglu B et al, 2016	Yes	Yes	Yes	Yes	No	No	Yes	Yes	Low
Atagun MI et al, 2018	Yes	Yes	Yes	Yes	Yes	Yes	Yes	Yes	Low
Boll KM et al, 2017	Yes	Yes	Yes	Yes	Yes	Yes	Yes	Yes	Low
Paval D et al, 2018	Yes	Yes	Yes	Yes	Yes	Yes	Yes	Yes	Low
